# Editorial: Case reports in anxiety and stress disorders

**DOI:** 10.3389/fpsyt.2025.1617174

**Published:** 2025-05-08

**Authors:** Elizabeth Stratton

**Affiliations:** ^1^ Central Clinical School, Faculty of Medicine and Health, The University of Sydney, Sydney, NSW, Australia; ^2^ Australian Research Council Centre of Excellence for Children and Families Over the Life Course, Sydney, NSW, Australia

**Keywords:** anxiety, stress disorder, PTSD, ligyrophobia, hikikomori, social anxiety

Anxiety and stress-related disorders are among the most common and debilitating mental health conditions globally, affecting an estimated 301 million people in 2019 alone, according to the World Health Organization (1). These conditions contribute significantly to years lived with disability (YLDs), reduce productivity, and impose a high burden on healthcare systems and individuals alike (2). Data from 23 community surveys conducted in 21 countries as part of the World Mental Health (WMH) surveys, involving over 50,000 participants, found that 9.8% had a 12-month DSM-IV anxiety disorder. Of those, only 27.6% received any form of treatment, and just 9.8% received care that could be considered possibly adequate. Furthermore, only 41.3% of individuals with a 12-month anxiety disorder recognized a need for care, with treatment rates being even lower in low-income countries (3). These findings underscore that standard diagnostic frameworks such as the DSM-5 or ICD-11 often fail to fully capture the variability in symptom expression across different populations, life stages, and cultural contexts, leading to misdiagnosis, undertreatment, or exclusion from existing care models (4).

There is growing evidence that individuals whose mental health experiences do not fit neatly within conventional psychiatric taxonomies often encounter significant barriers to recognition and effective care. Many individuals with atypical or culturally distinct mental health experiences face substantial barriers to recognition and care. Stigma, cultural shame, and lack of knowledge within both communities and healthcare systems often prevent people from seeking help or result in their symptoms being overlooked or misattributed, especially among marginalized groups (5). Although recent decades have seen advances in pharmacological treatments, psychotherapies, and digital interventions, significant gaps persist—particularly in delivering personalized, culturally sensitive, and contextually appropriate care. This is particularly crucial for those whose experiences do not fit neatly within conventional diagnostic categories. Research demonstrates that adopting flexible, evidence-based psychotherapies—those that preserve core treatment elements while adapting to individual needs and cultural contexts—improves outcomes, especially for underrepresented or complex cases (6).

Together, these four case report contributions reflect the power of context-sensitive, narrative-driven research to enrich psychiatric understanding and guide practice. They span clinical, cultural, and systemic domains, offering insight not only into what works, but how, why, and for whom. Importantly, two of the studies were conducted in non-Western, middle-income countries (Iran and Saudi Arabia), highlighting the global relevance of anxiety and stress research and the need to strengthen mental health systems worldwide.

Two original research articles set the stage with broader epidemiological and occupational perspectives. In “*Assessment of the relationship between health anxiety and Iranian nurses’ quality of life*”, Amoozadeh et al. examine how health anxiety adversely impacts the well-being of frontline nurses in Iran. Given the high stress and emotional labor involved in nursing—especially in post-pandemic contexts—this study underscores the need for systemic supports that address the psychological toll of healthcare work. The authors call for tailored interventions to mitigate anxiety and protect quality of life in caregiving professions.

In a similarly urgent vein, “*The prevalence of post-traumatic stress disorder among emergency medical services personnel in Saudi Red Crescent Authority, Riyadh*” by Alanazi et al. explores PTSD in emergency responders, a group routinely exposed to trauma. Their findings reveal strikingly high rates of PTSD symptoms, emphasizing the necessity of trauma-informed care structures within emergency services. The study provides evidence to support investment in preventive and rehabilitative mental health programs for Emergency Medical Service personnel—particularly relevant for low- and middle-income countries (LMICs), where mental health infrastructure is often underdeveloped or underfunded.

Adding depth and granularity to these population-level insights are two case reports that explore less common clinical presentations and therapeutic approaches. In “*Case report: Advances in treating ligyrophobia with third-generation ACT approach*”, Marino et al. detail the application of a third-generation Acceptance and Commitment Therapy (ACT) framework to treat a patient with ligyrophobia, a rare and debilitating fear of loud sounds. The report illustrates how modern therapeutic modalities—centered on values, psychological flexibility, and mindfulness—can be effectively adapted for idiosyncratic anxiety presentations that often elude standard protocols.

Even more striking in its implications for access and innovation is the report by Sakai et al., “*Successful remote treatment of a client with Hikikomori using internet-delivered cognitive therapy for social anxiety disorder*”. The case describes how a socially withdrawn individual, meeting the criteria for Hikikomori, was successfully treated via digital cognitive therapy. This intervention, grounded in evidence-based treatment for social anxiety disorder, was delivered entirely online. In addition to its clinical success, this report exemplifies the potential of telepsychology to overcome barriers to care—especially salient for LMICs, rural communities, and individuals for whom stigma, logistics, or cultural norms hinder access to mental health services.

Collectively, these articles underscore the need to move beyond one-size-fits-all solutions in the treatment of anxiety and stress-related disorders. They advocate for clinical and policy frameworks that integrate diversity—in symptoms, social conditions, and care modalities—into the very fabric of mental health care. This global lens is essential, especially in low- and middle-income countries (LMICs), where mental health resources are often limited, unevenly distributed, or burdened by stigma. The studies featured in this Research Topic serve as valuable exemplars of how interventions—whether digital, context-aware, or trauma-informed—can be effectively tailored to diverse populations and care settings. By drawing on real-world cases across regions and healthcare systems, and documenting both barriers and breakthroughs, these contributions illuminate the path toward more inclusive, scalable, and clinically rigorous models of care that are responsive to local needs and grounded in global relevance.

We hope this Research Topic inspires continued inquiry, collaborative learning, and practical innovation—paving the way toward more inclusive, equitable, and effective care for individuals experiencing anxiety and stress-related disorders, wherever they may be.
